# Supplementary material for: Sparse synaptic connectivity is required for decorrelation and pattern separation in feedforward networks
Source: Nat Commun. 2017 Oct 24;8:1116. doi: 10.1038/s41467-017-01109-y (PMC5653655; doi:10.1038/s41467-017-01109-y)
Supplement: Supplementary file 1 — Supplementary Information [file 41467_2017_1109_MOESM1_ESM.pdf]

Supplementary Information

Sparse synaptic connectivity is required for decorrelation and pattern separation in feedforward networks

N. Alex Cayco-Gajic, Claudia Clopath, R. Angus Silver

Supplementary Methods

Generation of Mossy Fiber activity patterns. To generate activity patterns with fixed mean and spatially-dependent correlations, we first defined the desired correlation coefficient of two mossy fibers (MFs) as a Gaussian function of the distance between them:

$$\rho(d) = Ae^{\frac{-d^2}{2\sigma^2}}$$

where d is the distance between the MFs, σ is the correlation radius, and A is a scaling factor. This yields a desired correlation matrix for the full population of MFs. We then used a Dichotomized Gaussian based method to generate binary vectors (representing population activity patterns) with the desired correlations and mean activity[1]. If the desired correlation is not positive semidefinite, an iterative correction procedure was used to find the nearest symmetric positive semidefinite matrix[2]. For these simulations, we chose the scaling factor A such that $\rho(0) = 0.9$ as this gave more stable fits than $\rho(0) = 1$. Similar results were obtained for lower values of $\rho(0)$. This procedure resulted in binary spike patterns with approximately the desired correlation coefficient matrix and the desired average f_{MF} (although the actual f_{MF} varied on pattern-by-pattern basis; see Fig. 1b, right).

Network Activity Dependent Thresholding (NADT). To model the effects of feedforward inhibition from Golgi cells, we used Network Activity Dependent Thresholding[3], which implements an adaptive threshold to increase the intrinsic threshold of a neuron by an amount proportional to the fraction of

active MFs (simulating the decrease in excitability due to feedforward inhibition). For the simplified model, NADT was implemented by modifying the threshold directly:

$$\theta = 3 + \alpha_{\mathrm{NADT}} f_{\mathrm{MF}}.$$

For the constant of proportionality, α_{NADT}, we took α_{NADT}=0.3, the previously experimentally estimated value[3]. In the biologically detailed model, NADT was implemented by increasing the tonic $GABA_A$ receptor-mediated conductance by a factor of $\alpha_{NADT} \cdot f_{MF}$. In practice, this meant incorporating this additional f_{MF} –dependent conductance into the leak conductance and leak reversal parameters of the integrate-and-fire model granule cell (GC).

Biologically detailed network model. We simulated a previously published model of the cerebellar input layer[3]. The model was written in NeuroML2 and simulated in jLEMS[4]. GCs were modeled as conductance-based integrate-and-fire model neurons with an absolute refractory period. A constant inhibitory conductance was included to model the effect of tonic $GABA_A$ receptor-mediated inhibition of GCs[5]. In practice, this meant that the $GABA_A$ receptor-mediated conductance and reversal potentials[6] were incorporated into the leak conductance and reversal potential parameters. Each excitatory synaptic conductance consisted of both AMPA receptor-mediated and NMDA receptor-mediated conductances that incorporate short-term plasticity[7,8] as well as double-exponential waveforms for direct and spillover components. A Mg^{2+} block was implemented by scaling the NMDAR conductances by a voltage-dependent function to model the fraction of unblocked NMDARs[9,10].

Finally, synaptic conductances were scaled by a factor of $1/N_{syn}$ to keep the maximum excitatory input to the GC fixed as the synaptic connectivity was varied. Parameters for synaptic waveforms and plasticity were fitted to previously published experimental data[3]. Passive parameters and parameters for the Mg^{2+} block mechanism were taken from previously published experiments[6,10,11].

Robustness. For a specified correlation radius, the robustness of GC learning, population sparsening, or expansion was defined as the fraction of parameter space (of synaptic connectivity and the fraction of active MFs) over which the normalized speed, or normalized population sparseness, or normalized total variance was >1 (e.g., GC learning was faster than MF learning). The robustness of decorrelation was defined as the fraction of parameter space over which the normalized population correlation was <1.

Heterogeneous variances. Because our definition of population correlation does not control for heterogeneities in the variance of different neurons, we reproduced our results after scaling the activity of each cell by its standard deviation over patterns. This scaling factor normalizes the variance of each cell to 1 to control for heterogeneous variances. However, the scaling did not change the qualitative effect of synaptic connectivity and mossy fiber (MF) input statistics on the population correlation. We therefore present the unscaled version in the manuscript for clarity.

Shuffling algorithm. The procedure for shuffling activity patterns was implemented as follows. First, to shuffle patterns from a higher population correlation to a lower population correlation, for each neuron i in the population we chose random patterns t_1 and t_2 and updated the patterns x by swapping the values of the neuron's activity:

$$x_i(t_1) \rightarrow x_i(t_2)$$
$$x_i(t_2) \rightarrow x_i(t_1)$$

This basic step was repeated over all neurons. This was then iterated until the population correlation of the shuffled activity patterns matched the desired value. Note that for an infinite number of iterations this reduces to traditional shuffling[12–15]. Iteration allows the partial removal of correlations rather than removing all correlations. Next, to shuffle patterns from a lower population correlation to a higher population correlation, we instead updated the patterns in the following way for all neurons:

$$x_i(t_1) \rightarrow \min(x_i(t_1), x_i(t_2))$$
$$x_i(t_2) \rightarrow \max(x_i(t_1), x_i(t_2))$$

This step modifies the activity patterns so that the neurons are together more active than their means in pattern t_2 and less active than their means in pattern t_1, thereby increasing the population correlation. This was iterated until the population correlation of the shuffled activity patterns matched the desired population correlation. Importantly, this algorithm changes the correlation structure without changing the single-cell statistics, including the variance and firing rates of each cell (Fig. 5b) nor the fraction of inactive cells (Fig. 5c). While it theoretically does change the population sparseness of the patterns

due to cross terms in the equation for population sparseness, in practice, the change was very small (Fig. 5c). While the second algorithm may introduce some higher-order correlations, because they are added in a stimulus-independent manner, they are unlikely to affect the information in the networks[16]. Because the algorithm is iterative, it was unable to converge for some parameter sets when the GC activity was extremely sparse (i.e., large N_{syn}, low f_{MF}), especially for the spiking model. These were discarded in the analysis.

Supplementary References

1. Macke, J. H., Berens, P., Ecker, A. S., Tolias, A. S. & Bethge, M. Generating spike trains with specified correlation coefficients. *Neural Comput.* **21,** 397–423 (2009).
2. Higham, N. J. Computing a nearest symmetric positive semidefinite matrix. *Linear Algebra Appl.* **103,** 103–118 (1988).
3. Billings, G., Piasini, E., Lorincz, A., Nusser, Z. & Silver, R. A. Network Structure within the Cerebellar Input Layer Enables Lossless Sparse Encoding. *Neuron* **83,** 960–974 (2014).
4. Cannon, R. C. *et al.* LEMS: a language for expressing complex biological models in concise and hierarchical form and its use in underpinning NeuroML 2. *Front. Neuroinform.* **8,** (2014).
5. Farrant, M. & Nusser, Z. Variations on an inhibitory theme: phasic and tonic activation of GABA(A) receptors. *Nat. Rev. Neurosci.* **6,** 215–229 (2005).
6. Seja, P. *et al.* Raising cytosolic Cl- in cerebellar granule cells affects their excitability and vestibulo-ocular learning. *EMBO J.* **31,** 1217–30 (2012).
7. Tsodyks, M. V. & Markram, H. The neural code between neocortical pyramidal neurons depends on neurotransmitter release probability. *Proc. Natl. Acad. Sci.* **94,** 719–723 (1997).
8. Tsodyks, M., Pawelzik, K. & Markram, H. Neural networks with dynamic synapses. *Neural Comput.* **10,** 821–835 (1998).
9. Woodhull, A. M. Ionic Blockage of Sodium Channels in Nerve. *J. Gen.*

Physiol. **61,** 687–708 (1973).

10. Rothman, J. & Silver, R. A. Data-driven modeling of synaptic transmission and integration. *Progress in molecular biology and translational science* **123,** 305–350 (2014).

11. Schwartz, E. J. *et al.* NMDA receptors with incomplete Mg^{2+} block enable low-frequency transmission through the cerebellar cortex. *J. Neurosci.* **32,** 6878–6893 (2012).

12. Nirenberg, S. & Latham, P. E. Population coding in the retina. *Current Opinion in Neurobiology* **8,** 488–493 (1998).

13. Panzeri, S., Golledge, H. D. R., Zhang, F. S., Tovee, M. J. & Young, M. P. Objective assessment of the functional role of spike train correlations using information measures. *Vis. cogn.* **8,** 531–547 (2001).

14. Panzeri, S. *et al.* The role of correlated firing and synchrony in coding information about single and separate objects in cat VI. *Neurocomputing* **44–46,** 579–584 (2002).

15. Nirenberg, S. & Latham, P. E. Decoding neuronal spike trains: how important are correlations? *Proc. Natl. Acad. Sci. U. S. A.* **100,** 7348–7353 (2003).

16. Cayco-Gajic, N. a., Zylberberg, J. & Shea-Brown, E. Triplet correlations among similarly tuned cells impact population coding. *Front. Comput. Neurosci.* **9,** (2015).

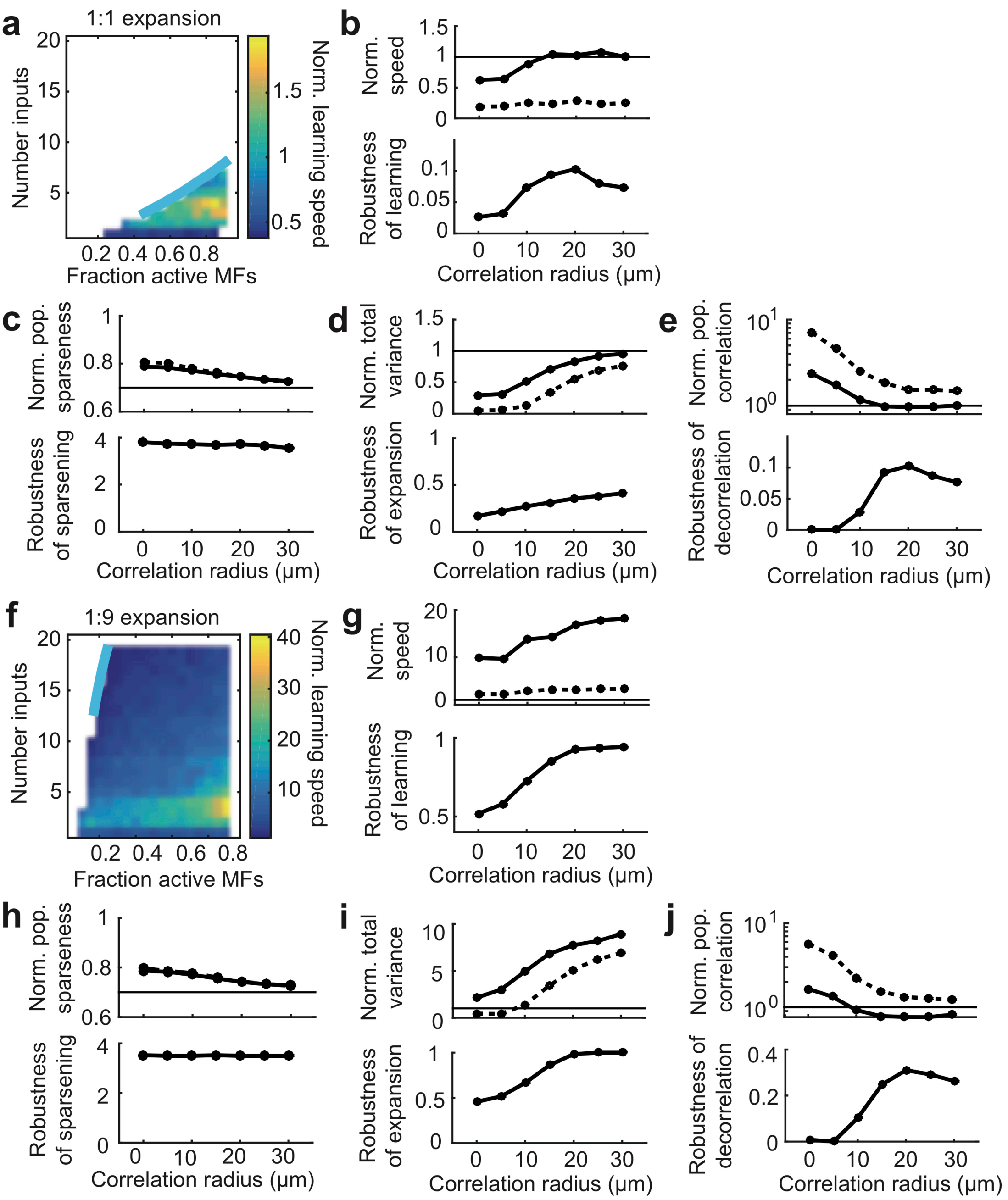

Supplementary Figure 1 I Varying expansion ratio does not qualitatively affect the dependence of pattern separation and learning on synaptic connectivity. (**a-e**) Learning speed and pattern separation for networks with a 1:1 expansion ratio. (**a**) Normalized learning speed for different fractions of active mossy fibers (MFs) and synaptic connectivities, for correlated MF inputs (σ = 20 μm). (**b)** Top: Median normalized learning speed (across f_{MF})

plotted for different correlation radii for sparse (N_{syn} = 4) and dense (dashed, N_{syn} = 16) synaptic connectivities. Bottom: Robustness of rapid granule cell (GC) learning for different correlation radii. (**c-e**) Same as (**b**) for normalized population sparseness (**c**), normalized total variance (**d**), and normalized population correlation (**e**). (**f-j**) Same as (**a-e**) but for a 1:9 expansion ratio.

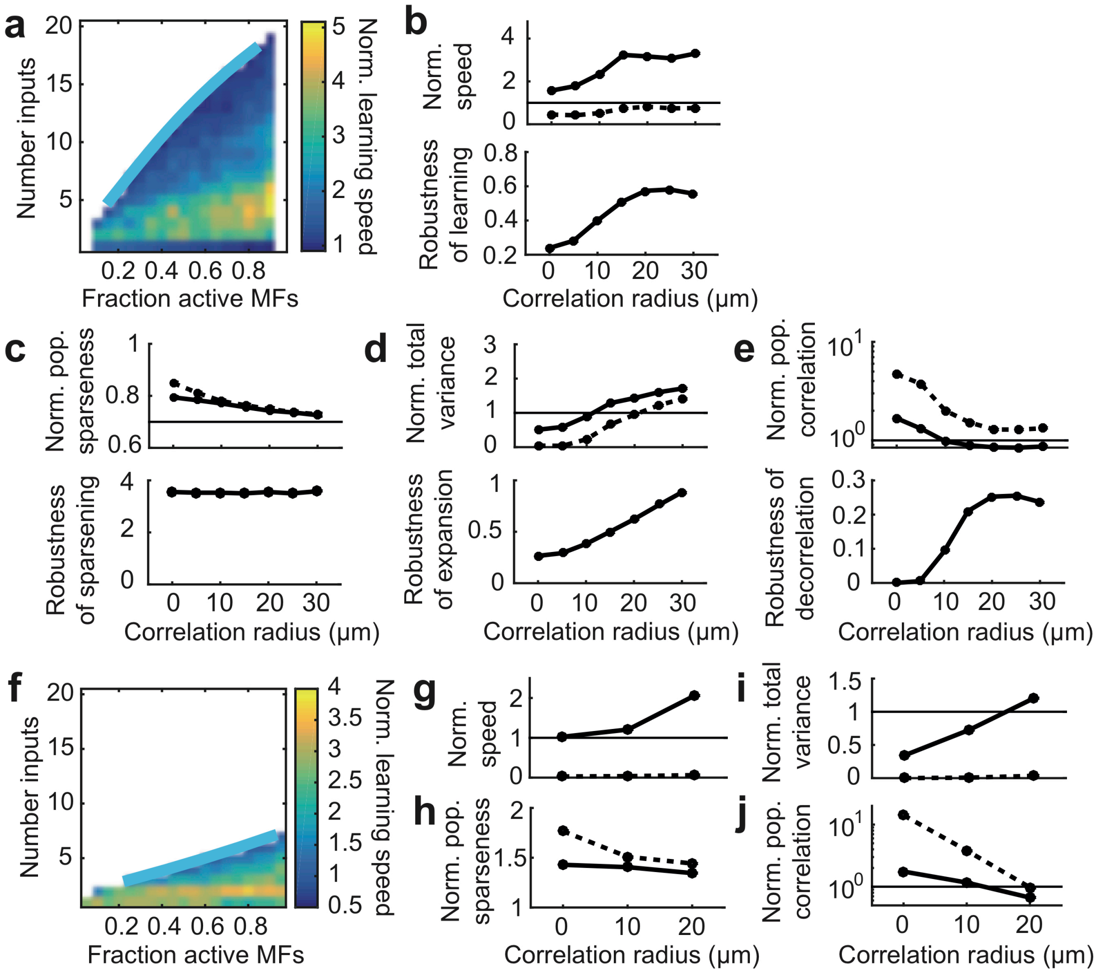

Supplementary Figure 2 I Network activity dependent thresholding does not qualitatively affect the dependence of pattern separation and learning on synaptic connectivity. (**a-e**) Learning and pattern separation in simplified networks with network activity dependent thresholding (NADT). (**a**) Normalized learning speed for different synaptic connectivities and fractions of active MFs, for correlated MF inputs (σ = 20 μm). (**b**) Top: Median normalized learning speed (across f_{MF}) plotted for different MF correlation radii for sparse ($N_{syn} = 4$) and dense (dashed, $N_{syn} = 16$) synaptic connectivities. Bottom: Robustness of learning of granule cell (GC) activity for different MF correlation radii. (**c-e**) Same as (**b**) for normalized population sparseness (**c**), normalized

total variance (**d**), and normalized population correlation (**e**). (**f-j**) Learning and pattern separation in biologically detailed networks with NADT. (**f**) Normalized learning speed for different synaptic connectivities and fractions of active MFs, for correlated MF inputs (σ=10μm). (**g**) Normalized learning speed plotted for different correlation radii for sparse ($N_{syn} = 4$) and dense (dashed, $N_{syn} = 16$) synaptic connectivities. (**h-j**) Same as (**g**) for normalized population sparseness (**h**), normalized total variance (**i**), and normalized population correlation (**j**).

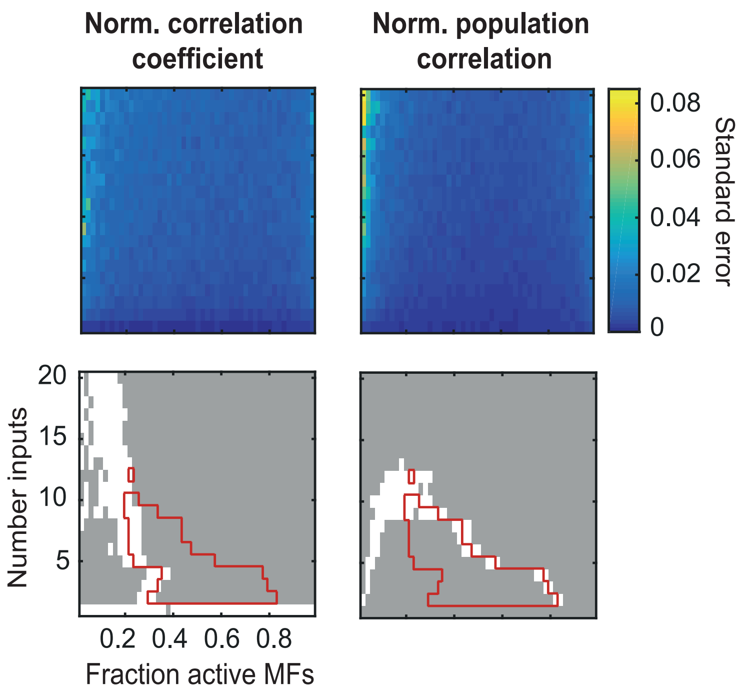

Supplementary Figure 3 I Statistical significance of decorrelation. Top row shows standard error of the normalized pairwise Pearson correlation coefficient (left) and normalized population correlation (right) for correlated mossy fiber (MF) inputs (σ = 20µm) with different f_{MF} and N_{syn} (cf. Fig. 3c,d for mean values). Bottom row shows parameter region in which there is a statistically significant difference between MF correlation and GC correlation (grey regions, Wilcoxon signed rank test with Bonferroni correction; white indicates lack of statistical significance), showing that the results are statistically significant over the majority of the parameter space. Red outline indicates the region in which the normalized population correlation <1 (corresponding to active decorrelation as described in the main text) but the normalized Pearson correlation coefficient >1; i.e., the red outline depicts the region over which the Pearson correlation coefficient incorrectly shows no active decorrelation.

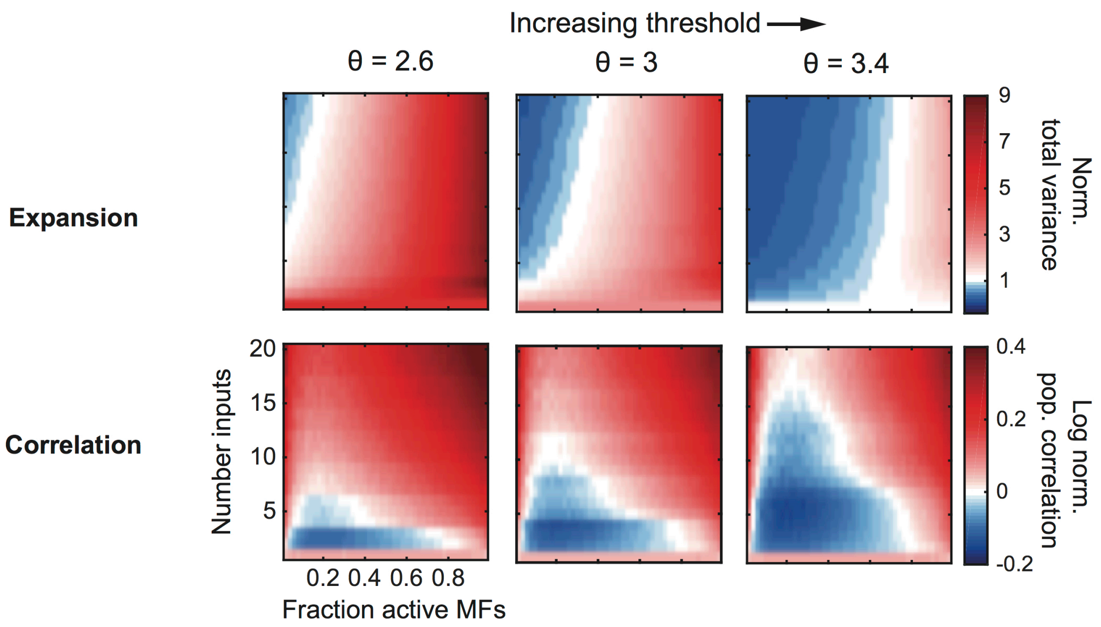

Supplementary Figure 4 l Increasing granule cell threshold increases decorrelation but decreases the size of the coding space. Top row shows relationship between normalized total variance for correlated mossy fiber (MF) inputs (σ = 20μm) with different synaptic connectivities and fractions of active MFs. Columns indicate increasing threshold, from left to right, where θ indicates the threshold. Bottom row as for top row but for log normalized population correlation.

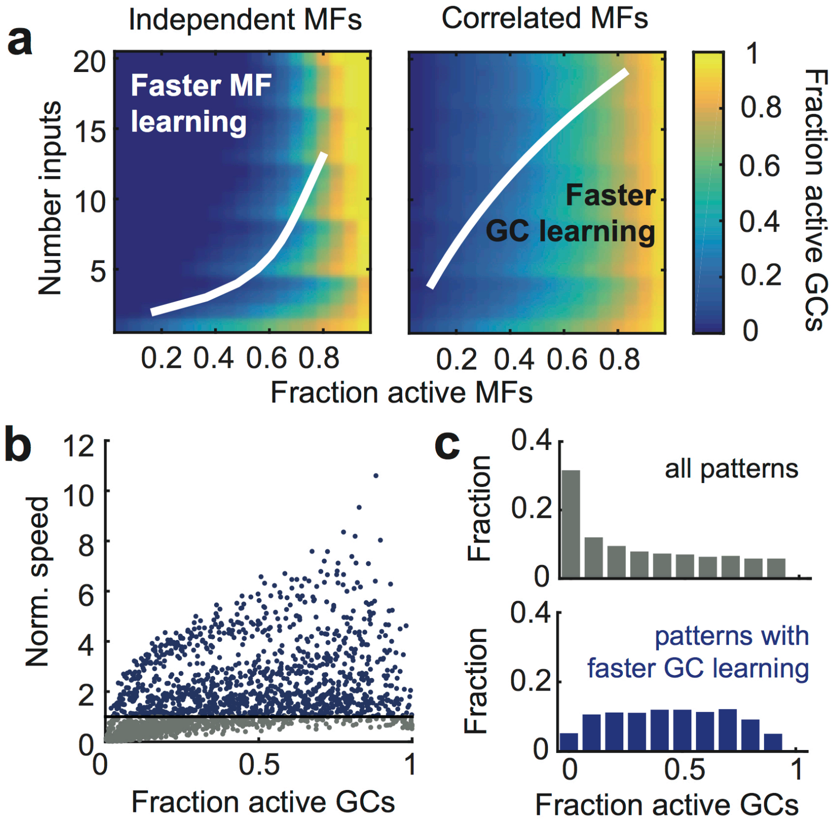

Supplementary Figure 5 I Pattern separation and decorrelation extends to regions of dense granule cell population activity. (**a**) Fraction of active granule cells (GCs; i.e., fraction of non-silent GCs, averaged over all activity patterns) for different synaptic connectivities and fractions of active mossy fibers (MFs), shown for both independent (left) and correlated (right, σ = 20 µm) MFs. White lines indicate boundaries at which the normalized learning speed equals 1 (see Fig. 1f). (**b**) Normalized learning speed plotted against the fraction of active GCs for all input statistics (i.e., the full range of f_{MF} and σ) and for all N_{syn}. Blue points indicate a normalized learning speed >1 (corresponding to faster learning and pattern separation) while grey points indicate a normalized learning speed <1. (**c**) Histograms of the fraction of active GCs over all patterns in the full parameter space (top; full range of N_{syn},

f_{MF}, σ) and over the region of parameter space in which the normalized GC speed >1 (bottom; corresponding to faster GC learning).

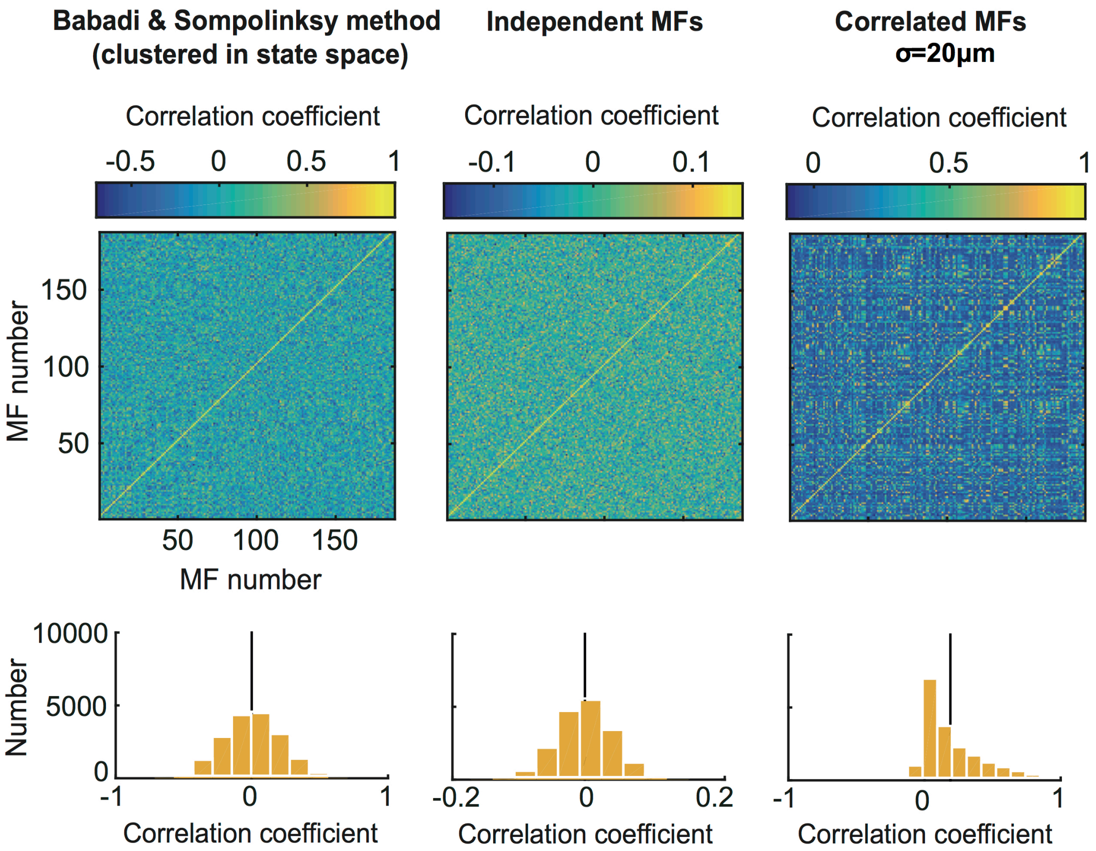

Supplementary Figure 6 I Comparison of clustered MF patterns. Top row shows correlation matrices for MF patterns generated by the method used in Babadi & Sompolinksy 2014 (left, clustered in state space) and the method used in this paper, both for independently activated MFs (center, not clustered), and spatially correlated MFs (right, clustered in physical space). Bottom row shows corresponding histograms of the correlation coefficient with mean values indicated in black. Note the different scale for the independent MFs.
